# Melanoma Following In Vitro Fertilization: Co-incident or Coincidence?

**DOI:** 10.7759/cureus.4857

**Published:** 2019-06-07

**Authors:** Philip R Cohen, Christof P Erickson, Brooke R Sateesh, Nathan S Uebelhoer, Antoanella Calame

**Affiliations:** 1 Dermatology, San Diego Family Dermatology, National City, USA; 2 Dermatology, Compass Dermatopathology, Inc., San Diego, USA; 3 Dermatologic Surgery, San Diego Family Dermatology, National City, USA

**Keywords:** clomiphene, estrogen, fertilization, infertility, in vitro, malignant, melanoma, post-partum, pregnancy, women

## Abstract

Melanoma may occur during or after natural or in vitro fertilization-associated pregnancy. A 43-year-old woman, who had received in vitro fertilization and developed a melanoma five months postpartum is described. Some studies have not shown in vitro fertilization to increase melanoma risk; however, several investigations have observed melanoma risk to be greater in women who have had this treatment. Therefore, although a potential increased risk for melanoma has been observed in infertile women who were either pregnant before or following in vitro fertilization, whether in vitro fertilization is an etiologic risk factor in the pathogenesis of melanoma for these individuals-or is merely a coincidental event-remains to be established.

## Introduction

The primary cause of melanoma is ultraviolet radiation from sun exposure or indoor tanning bed use [1-3]. Melanoma has been observed following delivery by natural pregnancy and in vitro fertilization-associated pregnancy [4-6]. A 43-year-old woman who had received in vitro fertilization and developed a melanoma five months after delivery of twins is described and the occurrence of post-in vitro fertilization melanoma is reviewed.

## Case presentation

A 43-year-old Fitzpatrick type 1 woman presented for evaluation of a mole that had changed in color and size on her left arm. She had no prior use of tanning beds and did not spend extensive time in the sun. Her past medical history was significant for primary infertility.

She received multiple cycles of in vitro fertilization treatments during the period of two years before conceiving. She became pregnant at the age of 42 years. Her medications included citrorelix acetate, estradiol, follitropin alpha, leuprolide acetate, and menotropins.

The early pregnancy was uneventful. However, at week 30 of gestation, the male fetus was noted to have an umbilical vein varix. Twice weekly nonstress test monitoring and weekly ultrasound were performed from week 30 of gestation until delivery.

She delivered healthy twins at 37 weeks and three days of gestation. Both the boy baby and the girl baby were normal with appropriate birth weight and no evidence of anomalies.

During the five months following delivery, she noticed changes in a pigmented lesion on her left arm. The lesion had been slightly raised and present since childhood. However, it had become larger not only in diameter but also in height.

**Figure 1:**
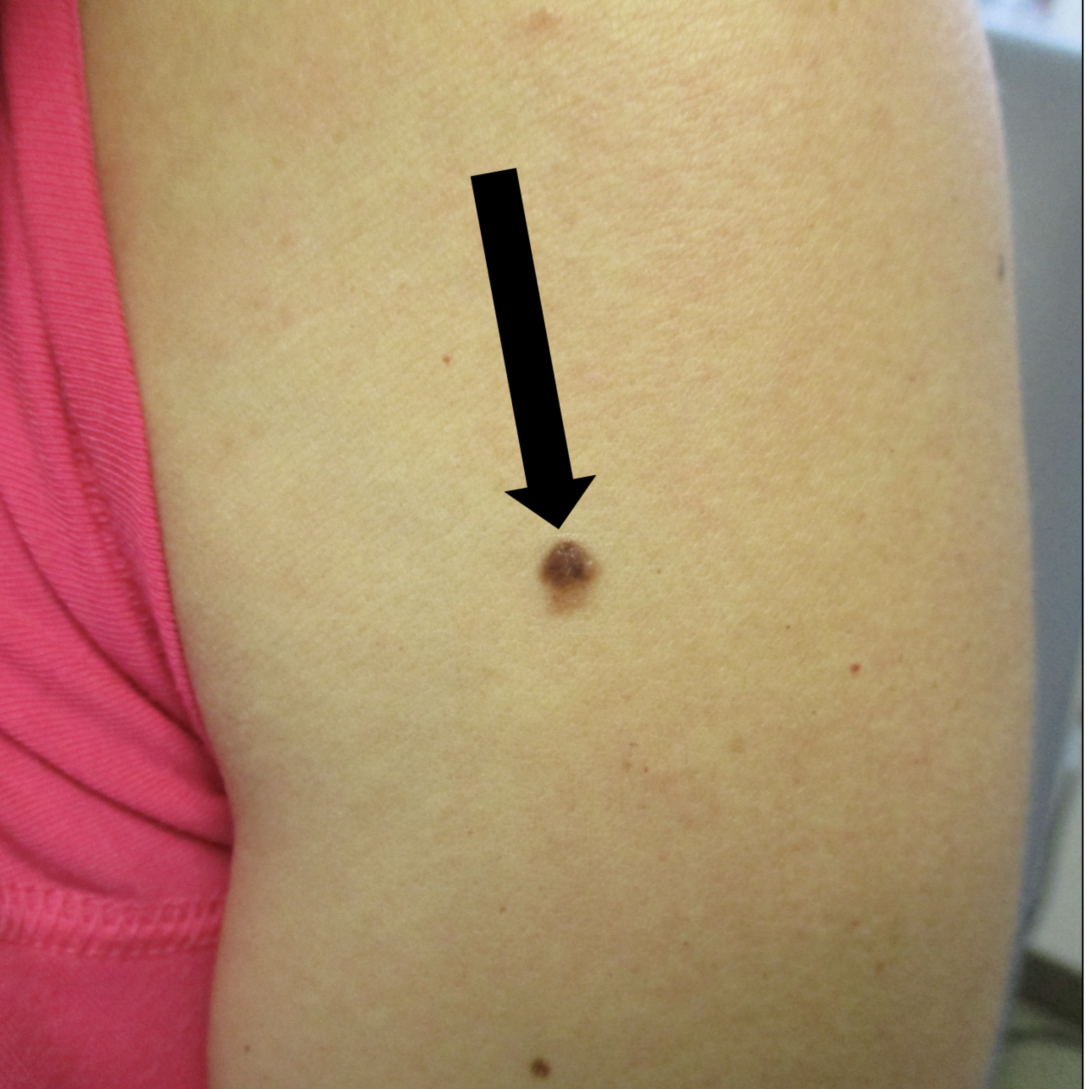
Malignant melanoma on the left proximal arm of a 43-year-old woman, which developed after she received multiple cycles of in vitro fertilization. The left proximal arm of a 43-year-old woman with primary infertility shows a malignant melanoma that developed within a previously benign compound nevus (black arrows). The melanoma appeared within five months after her delivery. She had received multiple cycles of in vitro fertilization treatments during a four-year period before conceiving.

Cutaneous examination showed a 5 mm x 5 mm multitoned brown papule and adjacent macule on the left proximal arm (Figure 1). There were no palpable axillary lymph nodes. A shave excision was performed.

**Figure 2:**
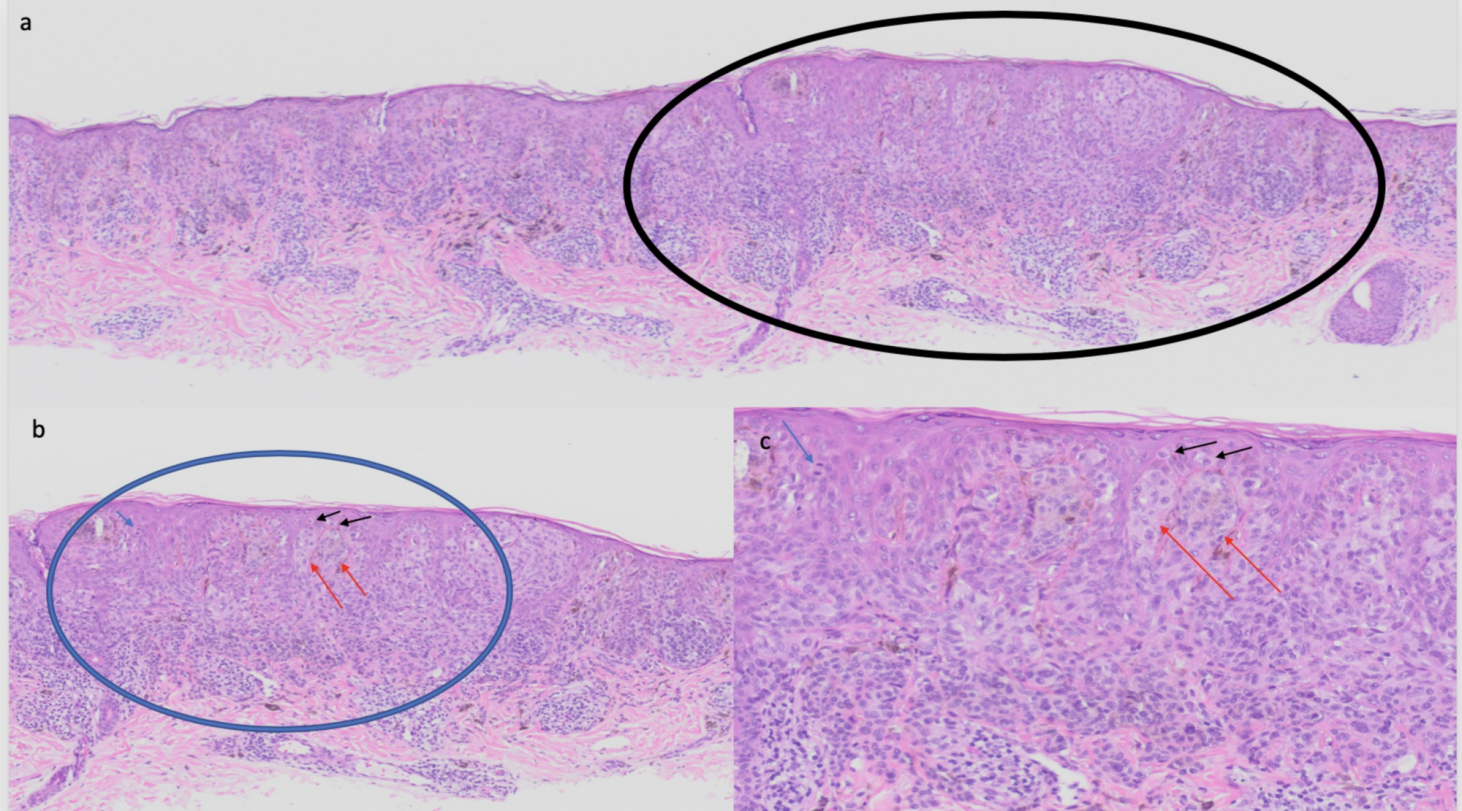
Pathology of post-in vitro fertilization melanoma: hematoxylin and eosin stained sections. Low (a) and higher (b and c) magnification views of the microscopic features of hematoxylin and eosin stained melanoma that developed in a compound nevus. The area circled in black (a) is shown in higher magnification (b); the area circled in blue (b) is shown in higher magnification (c). In addition to a benign compound nevus, there is an asymmetric proliferation of atypical melanocytes that appear as individual cells above the basal layer of the epidermis (black arrows) and as nests of melanocytes not only along the dermoepidermal junction but also in the dermis (orange arrows). A melanocyte in mitosis, found in the epidermis (blue arrow), is also present. The melanoma invades to a depth of 0.4 mm into the papillary dermis (hematoxylin and eosin stain: a, x2; b, x4; c, x 20).

Microscopic examination demonstrated benign appearing nests of melanocytes in the dermis and along the dermoepidermal junction; these changes were consistent with a compound nevus. Focally, within the compound nevus, there was an asymmetric proliferation of atypical melanocytes arranged in nests and individual cells at the dermoepidermal junction as well as in the upper layers of the epidermis; the atypical melanocytes also extended into the papillary dermis to a depth of 0.4 mm. A melanocyte in mitosis was noted in the epidermis; however, no dermal mitoses were seen. There was no evidence of ulceration or lymphovascular invasion (Figure 2).

**Figure 3:**
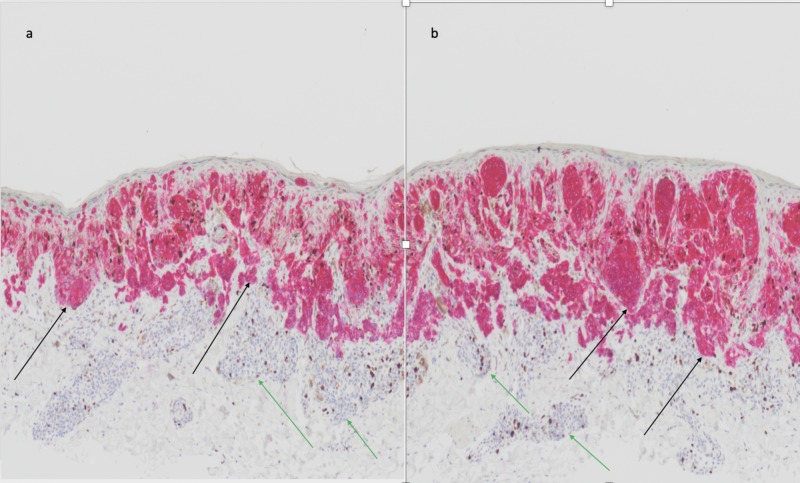
Pathology of post-in vitro fertilization melanoma: Ki-67 and MART-1 multiplex immunoperoxidase stained sections. Low (a) and higher (b) magnification views of the microscopic features of a Ki-67 and MART-1 multiplex immunoperoxidase stained melanoma that developed in a compound nevus. Ki-67 is a cancer antigen that is found in growing and dividing cells; its expression is strongly associated with cell proliferation and its presence reflects tumor aggressiveness. MART-1 (melanoma antigen recognized by T cells 1) is a melanosome-specific protein that results in cytoplasmic staining of all cells derived from melanocytes; however, it does not distinguish benign melanocytic nevi from melanoma. The combination Ki-67 and MART-1 stain, using a red chromogen to demonstrate the positive staining cells, highlights the tumor melanocytes of the malignant melanoma (black arrows) but does not stain the benign melanocytes of the compound nevus (green arrows) (Ki-67 and MART-1 multiplex immunoperoxidase stain: a, x10; b, x20).

Immunohistochemical studies were performed. Positive staining of the melanocytic proliferation within the dermal component was noted with a multiplex immunohistochemical stain for Ki-67 and MART-1 (Figure 3). HMB-45 staining highlighted not only the intraepidermal melanocytic proliferation but also focally stained the superficial dermal component.

Correlation of the clinical history and pathologic findings established the diagnosis of a malignant melanoma arising in a compound nevus. An excision of the site, with a one-centimeter margin, was performed. The surgical wound was repaired using a layered, side-to-side closure.

Complete skin examinations continue to be performed every three months. There has been no melanoma recurrence at follow-up visits every three months; also, no additional atypical-appearing pigmented lesions have been observed. The temporal association between the prolonged period of multiple in vitro fertilization cycles and the subsequent development of melanoma raises a consideration as to whether the melanoma is causally related to the infertility treatment. 

## Discussion

Approximately one-third of women who are diagnosed with melanoma are of childbearing age. Indeed, melanoma is the most common malignancy in women during pregnancy. In addition, the pregnant women in whom melanoma is diagnosed have an increased risk for not only recurrence, but also metastases and death [3-5].

Melanoma was traditionally considered to be a nonhormone-associated neoplasm. However, the influence of sex hormones (such as estrogen) and the presence of their receptors (such as estrogen receptor beta) on the tumor cells suggest that melanoma is indeed a hormone-sensitive malignancy. Specifically, melanoma growth and progression can be enhanced by decreased expression of estrogen receptor beta which normally seems to function as a tumor suppressor. However, the definitive mechanism of estrogen-associated melanoma carcinogenesis remains to be established; it might be postulated, in women following in vitro fertilization that the excess estrogen causes down regulation of estrogen receptor beta and thereby results in limiting the receptor-related suppression of melanoma [3-7].

In vitro fertilization, successfully initiated in 1978, results in supraphysiological levels of estrogen and progesterone. The major management aspects of in vitro fertilization include ovarian stimulation (which is also referred to as either controlled ovarian stimulation or controlled ovarian hyperstimulation) that utilizes either clomiphene citrate or gonadotropins (follicle stimulating hormone or recombinant human follicle stimulating hormone) or both and luteal phase stimulation (which is also referred to as luteal phase support) following oocyte retrieval that utilizes human chorionic gonadotropin or progesterone. In addition, some women also receive human menopausal gonadotropin to induce ovulation and/or gonadotropin releasing hormone agonists or antagonists either before or during ovarian stimulation [8-10].

There is an increased risk for breast, endometrial, and ovarian cancer in women with either delay or inability to achieve pregnancy. However, studies have also noted an increased risk of malignancy associated with in vitro fertilization; the use of fertility medications has been observed to modestly increase the risk of borderline ovarian cancer and high doses or multiple cycles of clomiphene citrate may increase the risk of endometrial cancer. However, breast, cervical, colon, and thyroid cancer do not appear to be more frequent in women who had prior in vitro fertilization [11-12].

Melanoma risk has been found to be higher in nulliparous women [13]. However, most retrospective studies evaluating the occurrence of melanoma after the use of fertility drugs have not shown an increase risk of this malignancy [11-13]. Yet, melanoma has occurred in women following in vitro fertilization [14]; indeed, melanoma risk has been noted to be greater in women aged 30 years and older-as compared to women less than 30 years of age-at first birth following in vitro fertilization [13].

Whether the fertility treatment is a contributing etiologic factor to the development of melanoma is a coincidental event still remains to be determined. One group of investigators found an increased risk of melanoma in women who had in vitro fertilization and gave birth as compared to women who remained nulliparous following in vitro fertilization [15]. In addition, another group of researchers noted that there was an apparent-albeit statistically nonsignificant-increased risk of melanoma associated with a higher number of invitro fertilization cycles, similar to our patient [13].

Some studies have noted melanoma risk to be increased after the use of clomiphene citrate [16-18]. Also, researchers of a large cohort study of women with infertility observed a significant increased risk for melanoma among parous patients who received gonadotropins or gonadotropin-releasing hormone [19]. However, some of the investigators noted a reduced risk of melanoma in women who had more than one cycle of either clomiphene citrate or human menopausal gonadotropin as compared to those who had one or no cycles [20].

A collaborative review of the literature regarding melanoma risk after in vitro fertilization was recently performed by researchers from multiple centers. They observed that the studies did not demonstrate a definitive association between the development of melanoma and prior in vitro fertilization among all infertile women. However, they also noted that the data revealed that ever-parous women with infertility who were treated with in vitro fertilization had a potential increased risk for melanoma [6]. 

## Conclusions

Melanoma occurs not only during but also following natural or in vitro fertilization-related pregnancy; indeed, several investigations have observed melanoma risk to be greater in patients who have experienced this fertility treatment. Similar to our patient, increased risk of melanoma after in vitro fertilization was noted in women who were successfully treated, women who received a higher number of treatment cycles, women who were treated with either clomiphene citrate or gonadotropins or gonadotropin-releasing hormone, and women aged 30 years and older at first birth. Hence, although there seems to be a potential increased risk for melanoma in ever-parous infertile women following in vitro fertilization, it still remains to be definitively determined whether in vitro fertilization actually creates a greater melanoma risk-or is merely a coincidental event-for these individuals.
